# Network nanostructured polypyrrole hydrogel/Au composites as enhanced electrochemical biosensing platform

**DOI:** 10.1038/srep11440

**Published:** 2015-06-15

**Authors:** Qinfeng Rong, Hongliang Han, Feng Feng, Zhanfang Ma

**Affiliations:** 1 Department of Chemistry, Capital Normal University, Beijing 100048, China

## Abstract

In this work, a new network nanocomposite composed of polypyrrole hydrogel (PPy hydrogel) loaded gold nanoparticles (AuNPs) was prepared. The PPy hydrogel was directly synthesized by mixing the pyrrole monomer and phytic acid, and the mixed solution can be gelated to form hydrogel at once. The three-dimensional network nanostructured PPy hydrogel not only provided a greater effective surface area for increasing the quantity of immobilized biomolecules and facilitated the transport of electrons and ions, but also exhibited an improved conductivity. Meanwhile, the electrodeposited AuNPs on the PPy hydrogel can further increase the specific surface area to capture a large amount of antibodies as well as improve the capability of electron transfer. The network PPy hydrogel/Au nanocomposites were successfully employed for the fabrication of a sensitive label-free amperometric immunosensor. Carcinoembryonic antigen (CEA) was used as a model protein. The proposed immunosensor exhibited a wide linear detection range from 1 fg mL^−1^ to 200 ng mL^−1^, and an ultralow limit of detection of 0.16 fg mL^−1^ (S/N = 3), and it also possessed good selectivity. Moreover, the detection of CEA in ten human serums showed satisfactory accuracy compared with the data determined by ELISA, indicating that the immunosensor provided potential application for clinical diagnosis.

Currently, how to enhance the performance of the electrochemical biosensor is still the main focus of biosensor research. For an electrochemical biosensor, its performance is closely related with the characteristics of the electrode interface^1^,^2^,^3^,^4^. Large specific surface area, high electrical conductivity, and excellent biocompatibility are the highly desirable properties^5^,^6^,^7^,^8^. It is because that a larger interfacial area can immobilize more biomolecules and increase the biometric identification probability. Excellent conductivity can facilitate electron transfer between the electrode and electrolyte, thus enhancing the sensitivity of the electrode. Good biocompatibility can keep the activity of biomolecules to much extent. In order to realize this goal, application of three-dimensional (3D) electrodes in electrochemical biosensors has received great attentions in recent years due to their many desirable properties^9^,^10^,^11^. For example, 3D graphene foam electrochemical electrode has been developed to detect dopamine due to their large surface area, low mass density, high electrical conductivity, and outstanding mechanical stability^12^. However, the 3D graphene foam grown by chemical vapor deposition is defect-free and highly hydrophobic, which imposes difficulties in its surface modification and biological applications. And its synthesis always involves complicated process, strict experimental conditions, and hard to directly fix on the surface of electrode. Therefore, a new type of electrode modified 3D nanomaterial is expected to be found, which could not only maintain excellent conductivity, but also can be simple preparation and modification.

Recently, conducting polymer hydrogels have been used as an alternative platform for construction of biosensor^13^,^14^,^15^. Particularly, nanostructured conducting polymer hydrogels not only retain the unique properties of conducting polymers, but also possess the characteristics of nanomaterials such as large surface area, quantum effect, and especially the 3D continuous conducting network, which further enhance the merit of conducting polymer hydrogels in designing and making novel biosensors^16^,^17^. These 3D nanomaterials have the potential to provide a number of advantages as follows: (1) large specific surface area are advantages over 2D nanosheets, 1D nanowires or 0D nanoparticles for electrochemical sensing applications due to large open channels on the nano-scale pores within the 3D interconnected porous structures; (2) the excellent electronic properties of conducting polymers due to their long π-conjugated backbone facilitate the rapid electron transfer; (3) the good biocompatibility of hydrogels promotes the immobilization of biomolecules and preserves their bioactivity; in addition, (4) conducting polymer hydrogels also provide excellent processability, which can be easily cast into thin film and any desired shapes as its gelation. No surfactants or templates are required, resulting in a much simple synthetic procedure. These attractive properties generate great interest for the field of electrochemical biosensor for the possible implementation of hygrogel in the constructing of electrode interfaces. Gold nanoparticles (AuNPs) have attracted considerable scientific interest because of their broad application in preparing biosensor, such as carriers of biomolecules and enhancement of the conductivity and the affinity to bioactive materials^18^,^19^. Those properties have promoted AuNPs as one of the most promising matrices applied in biomolecules immobilization. In recent years, electrodeposition of AuNPs has been demonstrated as a facile and rapid method with controllable thickness tightly attaching to the electrode surface^20^,^21^,^22^. Additionally, AuNPs can conjugate with biomolecules easily and can further increase the composite’s surface area and electrical conductivity^23^,^24^. Thus, electrodeposition of AuNPs could provide a facile and universal way to prepare functional biocomposite films on various conductive substrates for the development of biosensor.

In this work, we present the preparation of novel conducting polymer hydrogel (polypyrrole hydrogel)/AuNPs electrodes. The high porosity of the polypyrrole hydrogel (PPy hydrogel) is achieved by a simple preparation method using pyrrole as monomer and phytic acid as the gelatinizer and dopant to synthesize a nanometer scale conducting network. The electrode was prepared by electrochemical deposition an AuNPs layer onto surface of PPy modified glassy carbon electrode. AuNPs were integrated into the sensor and acted as enhanced materials to increase the electrical conductivity and promote electron transfer, further improving the sensitivity of the prepared biosensor. Additionally, the 3D nanostructured PPy hydrogel played important roles in the dispersion of AuNPs which acted as the biomolecule immobilization matrix and provided an electronically continuous 3D path for efficient charge collection. Combining with the advantages of PPy hydrogel and AuNPs, a sensitive label-free amperometric biosensor has been designed successfully. Carcinoembryonic antigen (CEA) was chosen as a model biomolecule. The prepared electrochemical biosensor exhibits superior performance for CEA detection with low detection limit, wide detection range, high sensitivity, and good stability.

## Results and discussion

**Figure 1:**
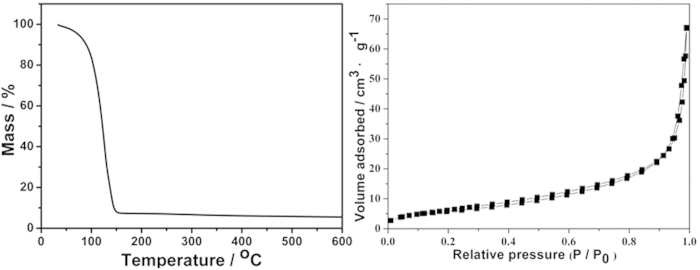
Thermal gravimertic analysis of PPy hydrogel (**A**) and Nitrogen adsorption–desorption isotherm of dehydrated PPy hydrogel.

In this work, we used phytic acid as the gelatinizer and dopant, pyrrole as monomer and ammonium persulfate as initiator to form PPy hydrogel. The above two kinds of solution mixed and gelated to form hydrogel at once, indicating that the polymerization is near instantaneous completion. Then a highly porous 3D nanostructure achieved on a large-scale and with long-term structural stability (Fig. S1). Using phytic acid as gelatinizers can render PPy hydrogel capable to possess excellent electronic property and good biocompatibility. The obtained PPy hydrogel owned a higher conductivity of 0.46 s cm^−1^, which was four times more than that of the other conducting polymer hydrogel^25^. After polymerization and purification through extensive rinsing with ultrapure water, the PPy hydrogel was subsequently swollen and its water content was measured to be 94.5% (w/w) by thermogravimetry (Fig. 1A). The specific surface area of dehydrated hydrogel was measured through Brunauer-Emmett-Teller was 26.2 m^2^ g^−1^ (Fig. 1B).

**Figure 2:**
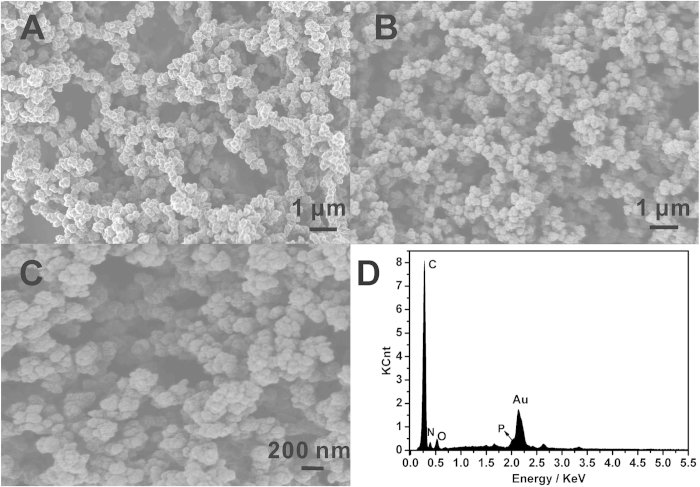
SEM image of PPy hydrogel (**A**) and different magnification of PPy hydrogel/Au composite (**B**) and (**C**), and (**D**) EDX of PPy hydrogel/Au composite.

The scanning electron microscope (SEM) images in Fig. 2A clearly revealed the 3D porous morphology of the dehydrated PPy hydrogel, and the hydrogel was composed of small PPy spheres stuck to each other. The 3D porous structure of polypyrrole hydrogel was induced by phytic acid which acted as crosslinkers. Because each phytic acid molecule could interact with more than one PPy chain, this cross-linking effect resulted in the formation of a mesh-like network. The porous nanoscale framework provided a greater effective surface area for increasing the quantity of immobilized biomolecules and facilitated the transport of electrons and ions. Moreover, the numerous pores within the PPy hydrogel could hold a large amount of water, which offered additional effective surface area between the PPy chains and solution phase as well as the enhanced conductivity of PPy hydrogel. Figure 2B,C are the SEM images of AuNPs electrodeposited on the 3D PPy hydrogel surface. In contrast to the smooth PPy hydrogel, the rough surface morphology of 3D PPy hydrogel/Au after electrodeposition demonstrates the formation of AuNPs film. The AuNPs can further increase the composite’s surface area and electrical conductivity, and can immobilize lots of antibodies and increase the immunoreaction probability and further enhance the sensitivity. The Energy dispersive X-ray (EDX) spectrum shows the compositions of PPy hydrogel/Au composite (Fig. 2D). Five major elements were shown in the sample: C, N, O, P and Au. Obviously, Au came from the AuNPs, while C, N, O and P originated from the PPy hydrogel.

**Figure 3:**
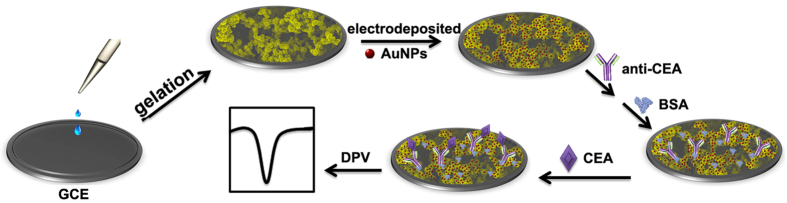
Schematic illustration of the electrochemical immunoassay protocol.

Early and accurate detection of specific target tumor markers at ultralow levels is utmost important for the ultimate control and therapy^26^,^27^,^28^. For an electrochemical biosensor, its performance is critically dependent on the properties of the chemical interface layer on electrode. The structure, specific surface area, biocompatibility, and electrical conductivity of the chemical interface layer play significant roles on the interface electrochemical signal transduction. Based on the excellent properties of the PPy hydrogel/Au composite, we successfully used it for fabricating a sensitive label-free amperometric biosensing platform with CEA as a model analyte, obtaining higher sensitivity superior to other platforms. For this immunosensor, the schematic illustration of the stepwise fabrication process was shown in Fig. 3. The 3D nanostructured PPy hydrogel can be gelated on the surface of glassy carbon electrode (GCE) directly. In order to further increase the surface area, electrical conductivity, and the ability of immobilizing antibody, AuNPs were electrodeposited on the surface of PPy hydrogel modified GCE. Anti-CEA was adsorbed onto the surface of the PPy hydrogel/Au composite. The specific binding of anti-CEA and CEA could be detected directly by the decrease of the current response.

**Figure 4:**
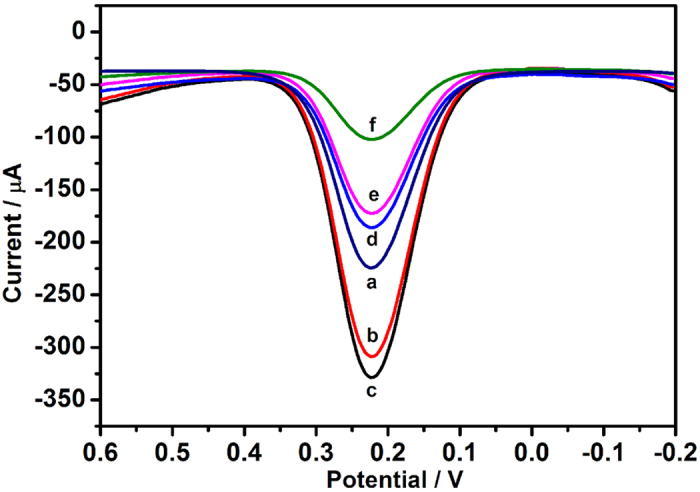
DPV responses of the modified procedure of electrodes in 0.01 M PBS containing 5.0 mM [Fe(CN)_6_]^4−/3−^ and 0.1 M KCl (pH 7.0) (**a**) bare GCE; (**b**) PPy hydrogel modified GCE; (**c**) Au/PPy hydrogel modified GCE; (**d**) anti-CEA/Au/PPy hydrogel modified GCE; (**e**) blocked with 1% BSA; (**f**) modified glassy carbon electrode after incubation with 1 ng mL^−1^ CEA.

The differential pulse voltammetry (DPV) was used to monitor the fabrication process and the performance of the immunosensor. As shown in Fig. 4, the current response of PPy hydrogel modified GCE (curve b) was higher than that of a bare GCE (curve a), which was attributed to the prominent electron transfer ability of PPy hydrogel. When AuNPs was electrodeposited on the PPy hydrogel surface, the current response further increased (curve c), exhibiting the excellent conductivity. In contrast, the loading of anti-CEA led to an obvious decrease of the peak current (curve d) owing to the formation of an electron-blocking layer. Subsequently, it was found that the current response further decreased after the immunosensor was blocked with BSA (curve e) and incubated in a solution with 1 ng mL^−1^ CEA (curve f). This may originate from the insulating BSA and CEA protein layers on the electrode that retards the electron transfer. Electrochemical impedance spectroscopy (EIS) was also used to measure the interfacial properties of the stepwise fabrication in different modification layers. The Nyquist plots of EIS consist of two portions: the linear part and the semicircle part. The linear portion at low frequencies represents the diffusion limited process and the semicircle diameter at the higher frequencies corresponds to the electron-transfer resistance. Figure S2 showed the Nyquist diagrams of the sensing electrode changed gradually with the stepwise modification processes. After Au/PPy hydrogel was modified on the GCE, the diameter of semicircle decreased significantly, revealing that the film of Au/PPy hydrogel can effectively promote the electron transfer (curve b). When stepwise immobilization of anti-CEA, BSA and CEA, the resistance increased gradually (curve c-e), attributing to the nonconductive layer of proteins hindered the electron transfer on the interface. Thus, the variation tendency of electron-transfer resistance of EIS plots verified the results of DPV plots in the same modification process.

**Figure 5:**
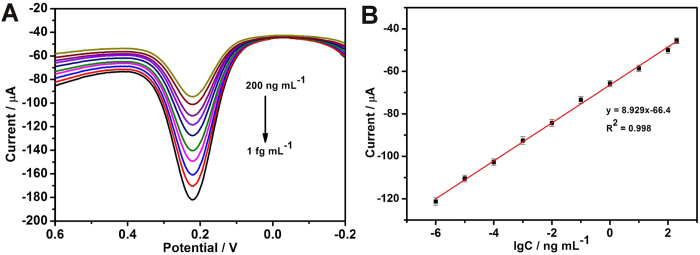
(**A**) DPV responses of electrochemical immunoassay in 0.1 M, pH 7.0 PBS containing [Fe(CN)_6_]^4−/3−^ (5 mM), curves a−i correspond to CEA at the concentrations from 0.01 fg mL^−1^ to 200 ng mL^−1^. (**B**) The calibration plot between the DPV peak current and the logarithm values of CEA concentrations. Error bars represent standard deviation, n = 3.

In order to achieve excellent analytical performance, the performances of the immunosensor on the factors of incubation time and the pH value of electrolyte were further investigated. The pH value of the electrolyte greatly affected the amperometric response of the immunosensor. In order to optimize the pH value, a series of PBS with the pH value ranging from 5.0 to 8.0 were studied. As shown in Fig. S3A, the experimental results show that the maximum amperometric response appears at about pH 7.0. Therefore, we chose the electrolyte of PBS with a pH 7.0 in the following tests. The incubation time for the immunoreaction also influence the response sensitivity of the fabricated biosensor. As obviously seen from Fig. S3B, the DPV current response of the immunosensor increased with the increasing incubated time until reached a plateau after 40 min, which revealed the saturated binding of the sandwich immunoreaction. Thus, the ideal incubation time was chosen to be 40 min for further investigation. Under the optimal conditions, the peak currents increased with increasing concentration of CEA. As shown in Fig. 5, the calibration plot displayed a good linear relationship between the peak currents and the analyte concentrations in the range of 1 fg mL^−1^ to 200 ng mL^−1^, and the equations can be expressed as I (μA) = 8.929 lgC (ng mL^−1^) – 66.4 with a correlation coefficient of 0.998. The limit of detection was estimated to be 0.16 fg mL^−1^ (S/N = 3). Compared with the previous reports (Table 1), it can be found that the immunosensor based PPy hydrogel/Au composite exhibited higher sensitivity, wider linear range and lower detection limit^29^,^30^,^31^,^32^. The exceptionally fast response and high sensitivity are attributed to the relatively short diffusion path that is due to the open channels of the network nanostructure and the continuously conductive framework of the PPy hydrogel/Au composite. AFP, UA, BSA, IgG, AA and glucose were used as interfering substances to investigate the specificity of the immunosensor. When the concentration of interfering substances was added for two order magnitude, the immunosensor exhibited similar current response toward the pure of CEA. Moreover, no obvious current response over the blank was observed for those nonspecific samples. These results indicated that the nonspecific reaction of the immunosensor toward the nonspecific proteins was negligible (Fig. S4). In addition, to evaluate the reliability and practical application of the proposed immunosystem, the CEA concentrations in human serum samples were measured and compared with ELISA as a reference method (Table 2). From the listed results, it was obvious to see that the relative error between the two methods ranged from −4.2% to 7.1%. These results indicated a good accuracy and acceptable reliability for CEA analysis in practical samples.

## Conclusion

In summary, a novel network nanostructured PPy hydrogel/Au composite was facilely synthesized and used as enhanced electrochemical biosensor platform. The superior properties of the nanocomposite include the following: (1) remarkable conductivity, the conductivity of PPy hydrogel is 0.46 s cm^−1^, which is superior to other conducting polymer hydrogel; (2) highly porous 3D continuous network, which promotes the transport of electrons, ions, and molecules, thereby benefiting subsequent electrochemical measurement; and (3) good biocompatibility, which promotes the immobilization of biomolecules and preserves their bioactivity. The PPy hydrogel/Au composite exhibited unprecedented performance and highly sensitivity as an electrochemical platform. Therefore, the PPy hydrogel/Au composite could be used as immunosensing platform for immunoassay, and holds unlimited potential for clinical diagnostics and other biosensor application.

## Methods

### Materials

Pyrrole, ammonium persulfate, phytic acid and BSA were bought from Sigma-Aldrich. Ascorbic acid, Hydrogen tetrachloroaurate hydrate (HAuCl_4_·xH_2_O, 99.9%), D-(+)-glucose and uric acid were achieved from Alfa Aesar (Tianjin, Cina). CEA, anti-CEA, AFP and IgG were purchased from Shanghai Linc-Bio Science Co., Ltd. (Shanghai, China). K_4_Fe(CN)_6_, K_3_Fe(CN)_6_, Na_2_HPO_4_, NaH_2_PO_4_ and KCl were obtained from Beijing Chemical Reagents Company (Beiing, China). Clinical human serum samples were provided by Capital Normal University Hospital (Beijing, China). All the chemical reagents were of analytical grade and used without further purification.

### Apparatus

All electrochemical measurements were carried out on a CHI832 electrochemical workstation (Chenhua Instrumentsn Co., Shanghai, China). SEM images and EDX were determined with a Hitachi SU8010 SEM. Thermogravimetry was analyzed on TGA/SDTA851 from METTLER TOLEDO. Ultrapure water used in all procedures was purified through an Olst ultrapure K8 apparatus (Olst, Ltd., resistivity >18 MΩ). A three electrochemical system in the experiment was composed of a GCE (4 mm in diameter) as the working electrode, a Platinum wire and an Ag/AgCl electrode as counter electrode and reference electrode, respectively.

### Synthesis of polypyrrole hydrogel

Solution A: 1 mL H_2_O and 0.5 mL phytic acid were mixed together, then 142 μL pyrrole was injected and sonicated 1 min. Solution B: 0.114 g ammonium persulfate was dissolved in 0.5 mL H_2_O. The solution A and B was cooled to 4 °C and then solution B was added into solution A quickly. The polypyrrole hydrogel was purified by dialysis (8000–14000 Mw cut off) for 3 days to remove by-products from polymerization and excess phytic acid. Finally, the obtained polypyrrole hydrogel was dried under vacuum at 60 °C.

### Fabrication of immunosensor

10 μL mixed solution of solution A and solution B was dropped on the surface of a pretreated GCE and allowed to polymerize for 15 min to form a thin homogeneous PPy hydrogel film. Subsequently, the electrode was immersed in ultrapure water for 20 minutes to remove excess ions and any oligomers. The PPy hydrogel modified electrode was obtained. The PPy hydrogel/Au composite was prepared by electrochemically deposited the AuNPs on the prepared PPy hygrogel/GCE by CV scanning from 0.2 V to −1.0 V in 0.5 mM HAuCl_4_ solution containing 0.1 M KCl at a scan rate of 50 mV s^−1^ for 10 cycles. After that, the modified electrode was rinsed with ultrapure water. Then, the PPy hydrogel/Au composite modified GCE was dipped in anti-CEA solution (200 μg mL^−1^ in 0.1 M PBS, pH 7.3) and incubated in a moisture-saturated environment overnight at 4 °C to absorb the antibodies. Finally, the resulting modified electrode was further incubated with a solution of BSA (1%, w/w) for 1 h at room temperature in order to block the remaining active sites against non-specific absorption. After every step, the modified electrode was thoroughly rinsed with ultrapure water and PBS (pH 7.3) to remove the loosely adsorbed species. The desired immunosensor was finally obtained and stored at 4 °C prior to use.

## Additional Information

**How to cite this article**: Rong, Q. *et al*. Network nanostructured polypyrrole hydrogel/Au composites as enhanced electrochemical biosensing platform. *Sci. Rep*. **5**, 11440; doi: 10.1038/srep11440 (2015).

## Supplementary Material

Supplementary Information

## Figures and Tables

**Table 1:** Comparison of linear range and detection limit of some modified electrodes materials.

Sensors	Linear range (ng mL^−1^)	Detection limit (ng mL^−1^)	Ref
Au/PDDA-EGO	1.0 × 10^−1^ to 200	5.0 × 10^−2^	29
Au/CS-PBGS	5.0 × 10^−3^ to 50	1.0 × 10^−3^	30
GNP-Thi-GS	1.0 × 10^−2^ to 0.5	4.0 × 10^−3^	31
Au-IL-GS	1.0 × 10^−6^ to 100	1.0 × 10^−7^	32
PPy hydrogel/Au	1.0 × 10^−6^ to 200	1.6 × 10^−7^	This work

**Table 2:** Determination of CEA in serum samples.

Sample	Proposed immunosensor (ng mL^−1^)	ELISA (ng mL^−1^)	Relative error (%)
1	0.91 ± 0.045	0.85	7.1
2	2.74 ± 0.086	2.86	−4.2
3	0.58 ± 0.021	0.56	3.6
4	4.79 ± 0.075	4.88	−1.8
5	1.37 ± 0.064	1.33	3.0
6	2.10 ± 0.036	2.03	3.4
7	1.27 ± 0.052	1.21	5.0
8	1.55 ± 0.046	1.50	3.3
9	1.41 ± 0.024	1.34	5.2
10	0.57 ± 0.013	0.59	−3.4

±Shows the standard deviation (n = 3).
