# Supplementary material for: Network nanostructured polypyrrole hydrogel/Au composites as enhanced electrochemical biosensing platform
Source: Sci Rep. 2015 Jun 15;5:11440. doi: 10.1038/srep11440 (PMC4466777; doi:10.1038/srep11440)
Supplement: Supplementary Information [file srep11440-s1.doc]

Supporting Information

**Network nanostructured polypyrrole hydrogel/Au composites as enhanced electrochemical biosensing platform**

Qinfeng Rong^1^, Hongliang Han^1^, Feng Feng, Zhanfang Ma*

Department of Chemistry, Capital Normal University, Beijing 100048, China


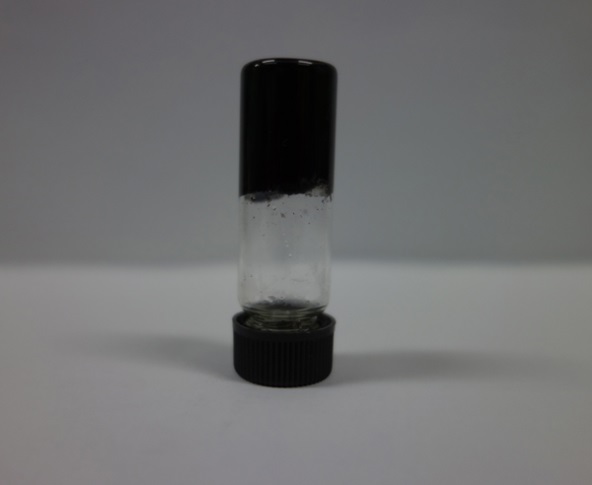


**Figure S1.** A photograph of the PPy hydrogel inside a glass vial.


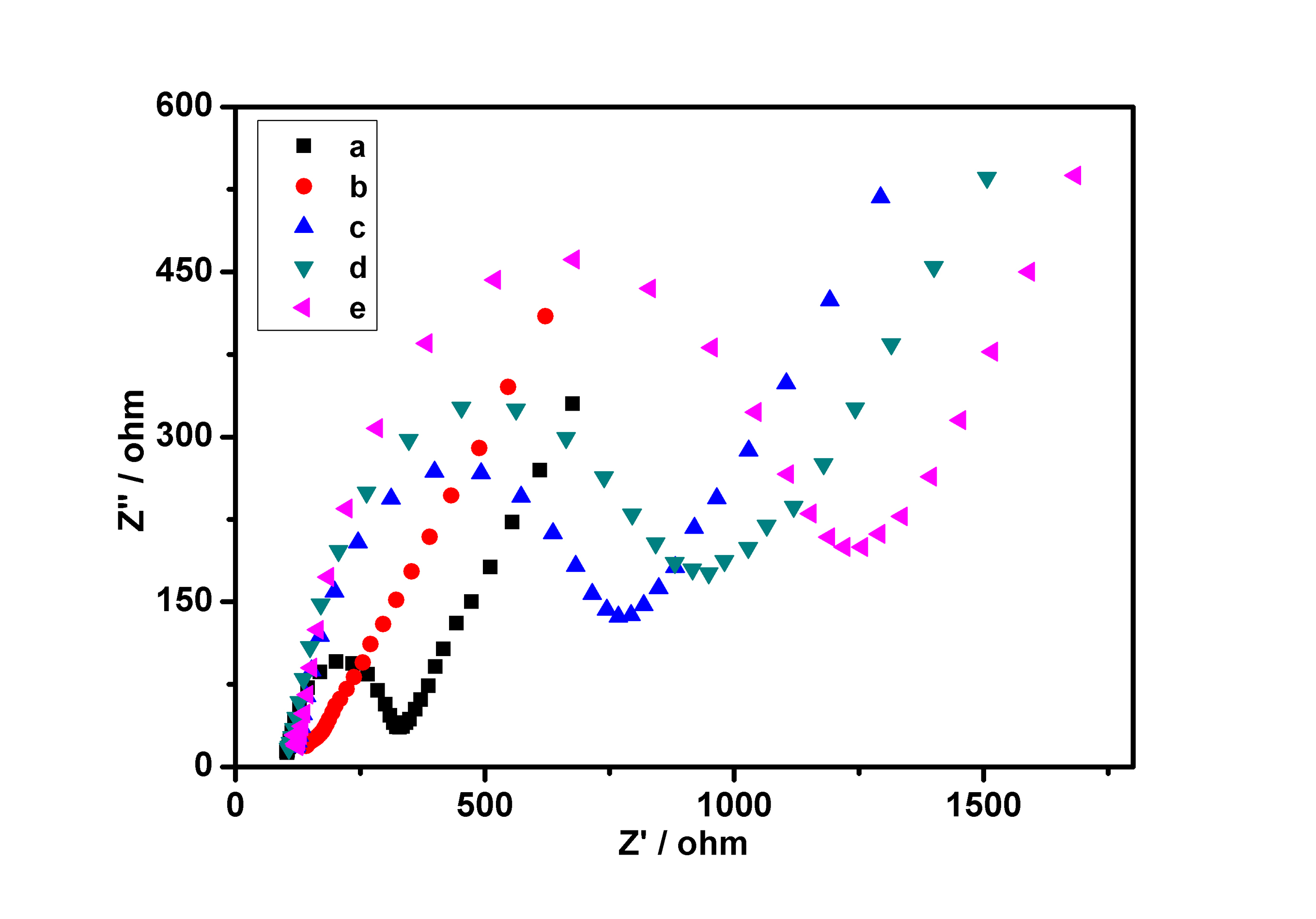


**Figure S2.** EIS measurements of the modified procedure of electrodes in 0.01 M PBS containing 5.0 mM [Fe(CN)_6_]^4-/3-^ and 0.1 M KCl (pH 7.0) (a) bare GCE; (b) Au/PPy hydrogel modified GCE; (c) anti-CEA/Au/PPy hydrogel modified GCE; (d) blocked with 1% BSA; (e) modified glassy carbon electrode after incubation with 1 ng mL^-1^ CEA.


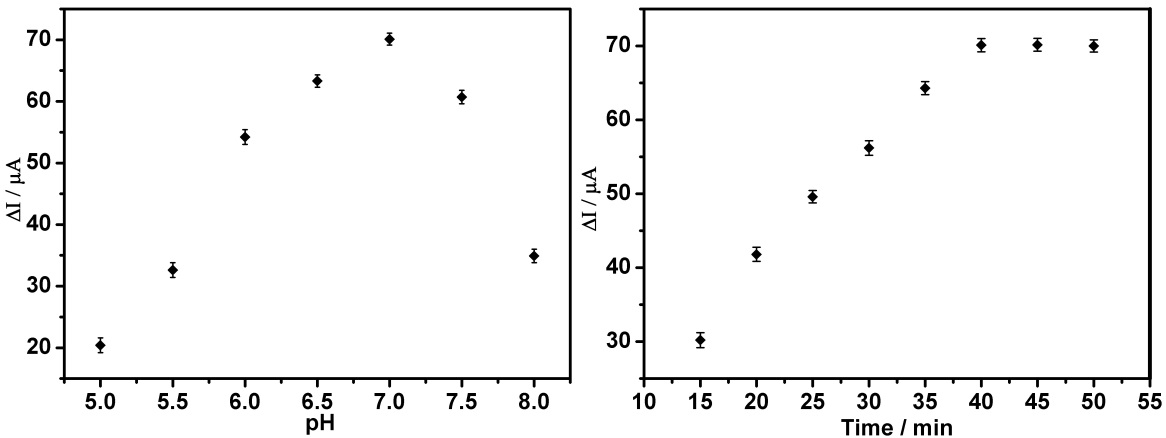


**Figure S3**. Effects of the (A) pH and (B) incubation time on the DPV responses of the immunosensor toward 50 ng mL^-1^ CEA.

**
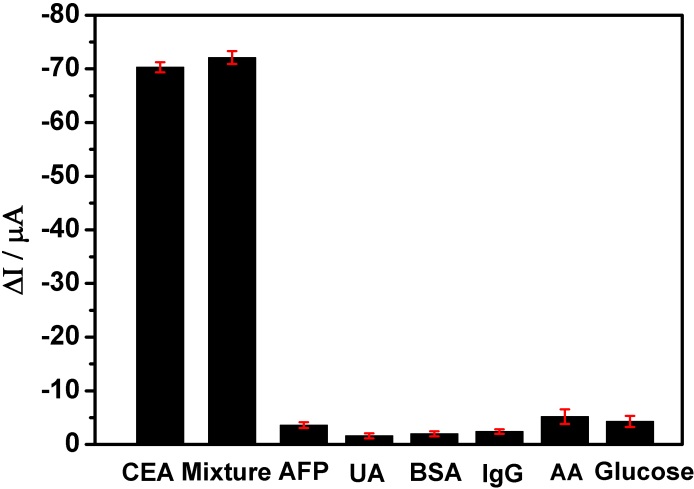
**

**Figure S4.** Current response of the immunosensor to 1 ng mL^-1^ carcinoembryonic antigen (CEA), mixture (1 ng mL^-1^ CEA+100 ng mL^-1^ alpha fetoprotein (AFP)+100 ng mL^-1^ uric acid (UA)+ 100 ng mL^-1^ bovine serum albumin (BSA)+100 ng mL^-1^ Human immunoglobulin G (IgG)+100 ng mL^-1^ ascorbic acid (AA)+100 ng mL^-1^ Glucose), 100 ng mL^-1^ AFP, 100 ng mL^-1^ UA, 100 ng mL^-1^ BSA, 100 ng mL^-1^ lgG, 100 ng mL^-1^ AA, and 100 ng mL^-1^ Glucose.
